# Effect of Porcine Epidemic Diarrhea Virus Infectious Doses on Infection Outcomes in Naïve Conventional Neonatal and Weaned Pigs

**DOI:** 10.1371/journal.pone.0139266

**Published:** 2015-10-06

**Authors:** Joseph T. Thomas, Qi Chen, Phillip C. Gauger, Luis G. Giménez-Lirola, Avanti Sinha, Karen M. Harmon, Darin M. Madson, Eric R. Burrough, Drew R. Magstadt, Holly M. Salzbrenner, Michael W. Welch, Kyoung-Jin Yoon, Jeffrey J. Zimmerman, Jianqiang Zhang

**Affiliations:** Department of Veterinary Diagnostic and Production Animal Medicine, Iowa State University, Ames, Iowa, United States of America; Virginia Polytechnic Institute and State University, UNITED STATES

## Abstract

Porcine epidemic diarrhea virus (PEDV) was identified in the United States (U.S.) swine population for the first time in April 2013 and rapidly spread nationwide. However, no information has been published regarding the minimum infectious dose (MID) of PEDV in different pig models. The main objective of this study was to determine the oral minimum infectious dose of PEDV in naïve conventional neonatal piglets and weaned pigs. A U.S. virulent PEDV prototype isolate (USA/IN19338/2013) with known infectious titer was serially ten-fold diluted in virus-negative cell culture medium. Dilutions with theoretical infectious titers from 560 to 0.0056 TCID_50_/ml together with a medium control were orogastrically inoculated (10ml/pig) into 7 groups of 5-day-old neonatal pigs (n = 4 per group) and 7 groups of 21-day-old weaned pigs (n = 6 per group). In 5-day-old pigs, 10ml of inoculum having titers 560–0.056 TCID_50_/ml, corresponding to polymerase chain reaction (PCR) cycle threshold (Ct) values 24.2–37.6, resulted in 100% infection in each group; 10ml of inoculum with titer 0.0056 TCID_50_/ml (Ct>45) caused infection in 25% of the inoculated pigs. In 21-day-old pigs, 10ml of inoculum with titers 560–5.6 TCID_50_/ml (Ct 24.2–31.4) resulted in 100% infection in each group while 10ml of inoculum with titers 0.56–0.0056 TCID_50_/ml (Ct values 35.3 –>45) did not establish infection in any pigs under study conditions as determined by clinical signs, PCR, histopathology, immunohistochemistry, and antibody response. These data reveal that PEDV infectious dose is age-dependent with a significantly lower MID for neonatal pigs compared to weaned pigs. This information should be taken into consideration when interpreting clinical relevance of PEDV PCR results and when designing a PEDV bioassay model. The observation of such a low MID in neonates also emphasizes the importance of strict biosecurity and thorough cleaning/disinfection on sow farms.

## Introduction

Porcine epidemic diarrhea virus (PEDV), the causative agent of porcine epidemic diarrhea (PED), is an enveloped positive-sense, single-stranded RNA virus belonging to the order *Nidovirale*, family *Coronaviridae*, subfamily *Coronavirinae*, genus *Alphacoronavirus* [1]. PEDV was identified in 1978 [2, 3] although the disease was first recognized in England in 1971 [4]. PEDV caused widespread epidemics in several European countries during the 1970s and 1980s [5, 6]; however, since the 1990s PED has become rare in Europe with occasional outbreaks [7]. PED was first reported in Japan in 1982 and has since been confirmed in other Asian countries such as South Korea, China, Thailand, and Vietnam [5, 8, 9]. PEDV was detected in the United States (U.S.) for the first time in April 2013 [10]. So far, at least two genetically different PEDV strains have been identified in the U.S. (U.S. PEDV prototype strain and S-INDEL-variant strain) [11, 12]. After the PED outbreak in the U.S., detection of U.S. prototype-like PEDV has been reported in Canada, Mexico, Taiwan, and South Korea [11, 13–15]; detection of U.S. S-INDEL-variant-like PEDV has been reported in South Korea, Germany, Belgium and France [16–20].

Since its emergence in the U.S., PEDV has spread rapidly across the country and resulted in the estimated death of over 7 million pigs in the first year [21], causing substantial economic losses. PED in U.S. swine is characterized by watery diarrhea, dehydration, variable vomiting, high mortality in neonatal piglets, and high morbidity but low mortality in weaned pigs [10]. The rapid spread of PEDV suggests that the virus is highly transmissible. However, no information has been published regarding the minimum infectious dose (MID; the smallest quantity of virus to establish infection) of PEDV in pigs at different stages of production.

Real-time RT-PCR (rRT-PCR) has been widely used for PEDV detection and diagnosis. However, correlations of PEDV infectious titers to the rRT-PCR cycle threshold (Ct) values have not been described. Virus isolation has generally been used to assess if a live virus is present in samples. But for PEDV, the success rate of virus isolation in cell culture has been quite low [22]. Currently, swine bioassay remains the most reliable means to determine if infectious PEDV is present in a clinical specimen or if "X" treatment will inactivate the virus. However, there remains inconsistency in selecting swine models for bioassay.

The objectives of this study were to 1) determine the minimum infectious dose of PEDV in naïve conventional neonatal piglets and weaned pigs; 2) determine the correlation of PEDV PCR Ct values to the infectious titers. Applications of these research data to the interpretation of the clinical relevance of PEDV diagnostic results and to development of a sensitive swine bioassay model for PEDV are discussed.

## Materials and Methods

### Virus and cells

A U.S. PEDV prototype strain cell culture isolate USA/IN19338/2013 was isolated and propagated in Vero cells (ATCC CCL-81) in our lab as previously described [22]. A virus stock at the 7^th^ passage in cell culture was prepared and used in this study. The virus stock was 10-fold serially diluted in post-inoculation media (Minimum Essential Medium supplemented with 0.3% tryptose phosphate broth, 0.02% yeast extract, 5 μg/ml of trypsin 250, 10 unit/ml penicillin, 10 μg/ml streptomycin, 0.05 mg/ml gentamicin, and 0.25 μg/ml amphotericin B) to obtain 10^−1^ to 10^−10^ dilutions (Table 1). The post-inoculation medium without virus was used as a negative control. Multiple aliquots of each dilution were prepared and stored at -80°C so that quantification of these virus dilutions and inoculation into pigs could be performed under the same number of freeze/thaw cycles.

**Table 1:** Infectious titers, PCR Ct values and genomic copies/ml of serial dilutions of PEDV stock.

Dilution of stock virus	TCID_50_/ml (Theoretical)	TCID_50_/ml (Back titrated)	PFU/ml (Back titrated)	Ct*	Genomic Copies/ml*
Stock Virus	562,000	562,000	680,000	12.2	2.10E+10
10^−1^	56,200	31,600	49,000	16.76	9.54E+08
10^−2^	5,620	5,620	6,600	20.21	9.20E+07
10^−3^	562	316	430	24.22	6.07E+06
10^−4^	56.2	56.2	23	28.22	4.03E+05
10^−5^	5.62	0	5	31.37	4.77E+04
10^−6^	0.562	0	0	35.28	3.37E+03
10^−7^	0.0562	0	0	37.62	6.89E+02
10^−8^	0.00562	0	0	>45	0
10^−9^	0.000562	0	0	>45	0
10^−10^	0.0000562	0	0	>45	0
Neg Ctl	0	0	0	>45	0

* Average values of triplicate PCR reactions for each dilution.

### Virus titration

The virus stock and serial dilutions were back titrated in Vero cells to determine their infectious titers in the unit of median tissue culture infective dose per milliliter (TCID_50_/ml) and plaque forming unit per milliliter (PFU/ml). To determine TCID_50_/ml, each sample was serially 10-fold diluted in post-inoculation media and inoculated into Vero cells grown in 96-well plates, 100μl per well, triplicate wells per dilution. The plates were incubated at 37°C with 5% CO_2_ for 5 days. Viral cytopathic effects (CPE) were recorded daily. After 5-day inoculation, the plates were subjected to immunofluorescence staining using a monoclonal antibody conjugate SD6-29 against the PEDV nucleocapsid protein (SD-1F-1, Medgene Labs, Brookings, South Dakota, USA). The virus titers were determined according to the method described by Reed and Muench [23] and expressed as TCID_50_/ml. To determine PFU/ml, each sample was serially 10-fold diluted in post-inoculation media and inoculated into Vero cells grown in 6-well plates, 500μl per well, duplicate wells per dilution. After incubation at 37°C for 1 h, 4ml of overlay medium (post-inoculation media supplemented with 0.75% carboxymethyl cellulose) was added to each well. The plates were then incubated undisturbed at 37°C with 5% CO_2_ for 4 days. The media were discarded and the plates were stained with crystal violet in 10% buffered formalin. The bottoms of the plates were then illuminated. Wells containing 20–200 plaques were counted and titers expressed as PFU/ml.

### RNA extraction

Nucleic acids were extracted from virus dilutions, rectal swabs, and tissue homogenates using a MagMAX Pathogen RNA/DNA Kit (Thermo Fisher Scientific, Waltham, Massachusetts, USA) and a Kingfisher-96 instrument (Thermo Fisher Scientific) following the instructions of the manufacturer.

### Quantitative real-time RT-PCR for PEDV

A PEDV nucleocapsid (N) gene-based real-time RT-PCR was previously developed at the Iowa State University Veterinary Diagnostic Laboratory (ISU VDL) [24, 25]. On the basis of the PEDV N gene-based rRT-PCR, a quantitative rRT-PCR to determine genomic copies/ml of PEDV in a sample was developed in this study.

To generate *in vitro* transcribed RNA standards, a fragment covering the PEDV N gene-based RT-PCR products (nucleotide positions 26,679–26,885 of the PEDV USA/IN19338/2013, GenBank # KF650371) flanked by restriction sites *EcoR*I and *Hind*III at its 5’ and 3’ end, respectively, was cloned into the plasmid vector pIDTBlue to obtain the plasmid pIDTBlue:PEDV_N_IVT (IDT, Coralville, Iowa, USA) in which the PEDV N gene products were located downstream of the bacteriophage T7 RNA polymerase promoter. The recombinant plasmids were transformed into One Shot® TOP10 chemically competent *Escherichia coli* cells (Thermo Fisher Scientific) and propagated following the instruction manual. The plasmids were extracted using a QIAprep® Spin Miniprep Kit (Qiagen, Valencia, California, USA) according to the manufacturer’s instructions. The plasmid DNA was linearized with *Hind*III, treated with Proteinase K, purified with QIAquick® PCR purification kit (Qiagen), and resuspended in nuclease-free water. The linearized DNA was subject to run-off *in vitro* transcription into RNA using a MEGAshortscript^™^ T7 Transcription Kit (Thermo Fisher Scientific) followed by purification using a MEGAclear^™^ Transcription Clean-up Kit (Thermo Fisher Scientific) according to the instruction manuals provided with the kit. The *in vitro* transcribed RNA was quantified using a BioSpectrometer (Eppendorf, Enfield, Connecticut, USA).

The *in vitro* transcribed RNA was 10-fold serially diluted in nuclease-free water and used as standards in the PEDV N gene-based rRT-PCR to generate standard curves and quantify viral loads in test samples. Five μl of each RNA template was used in PCR setup in a 25μl total reaction using Path-ID™ Multiplex One-Step RT-PCR Kit (Thermo Fisher Scientific) as previously published [24, 25]. Amplification reactions were performed on an ABI 7500 Fast instrument (Thermo Fisher Scientific, Waltham, Massachusetts, USA) with the following conditions: 1 cycle of 48°C for 10 min, 1 cycle of 95°C for 10 min, and 45 cycles of 95°C for 15 sec and 60°C for 45 sec. After generating 22 standard curves, the Ct values were averaged (means determined) and an equation of X = 10^(Ct-47.262)/-3.3969^, where X = genomic copies/ml, was developed to transform the Ct values into estimated genomic copies of PEDV RNA per ml in test samples under the PCR conditions of this study.

### Ethics statement

The experimental protocol for the pig studies was approved by the Iowa State University Institutional Animal Care and Use Committee (IACUC, Approval No. 4-14-7777-S; approved on 24^th^ of April 2014).

### Neonatal pig study design

**Fig 1:**
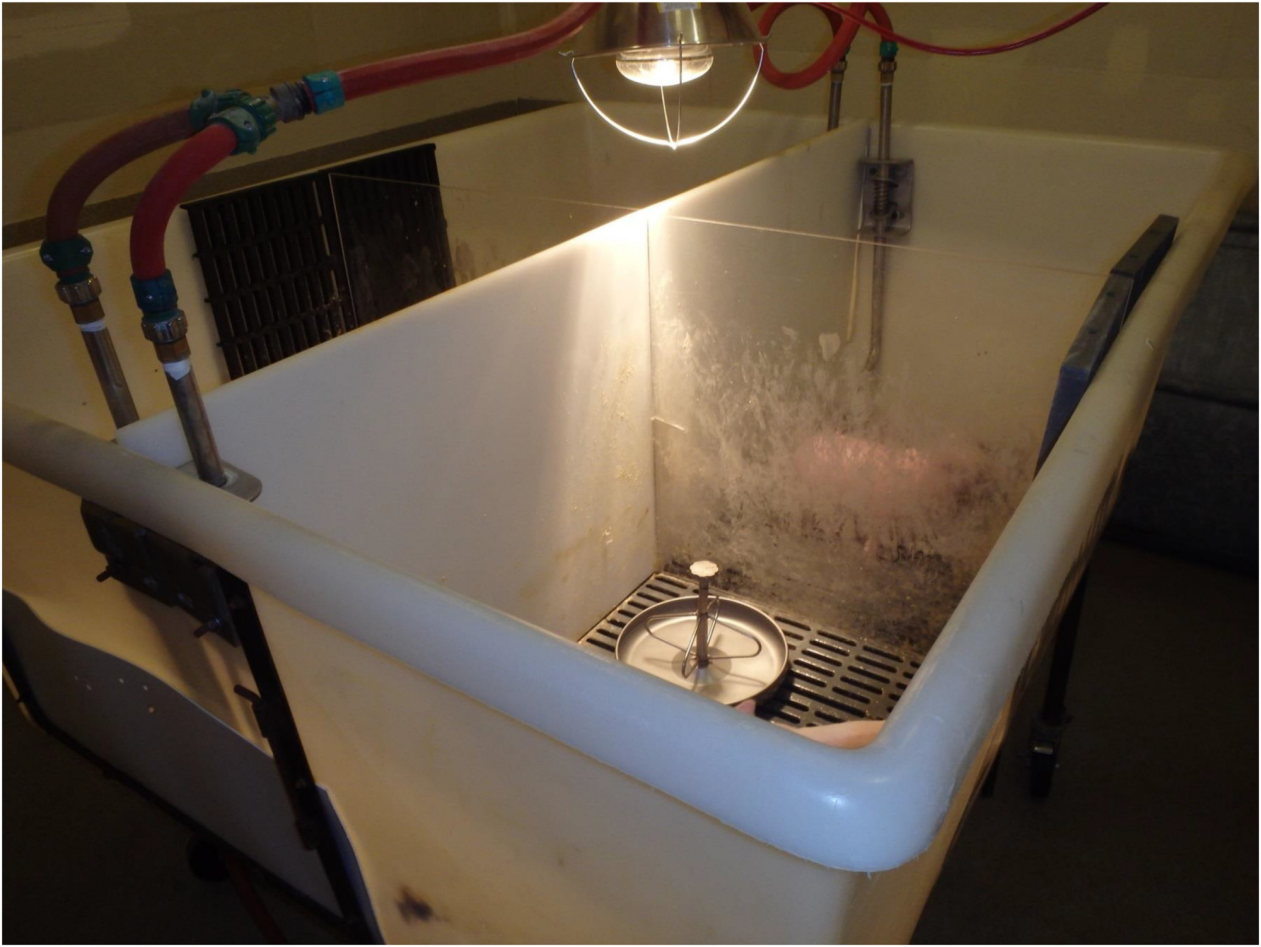
Elevated tubs used to house 5-day-old piglets in this study. One tub was located in each room. Each tub was split into 4 compartments with one pig per compartment. Design of the tub prevented contact between pigs and movement of feces or other waste between tub compartments.

Twenty-eight 5-day-old piglets purchased from a conventional breeding farm were tested at the ISU VDL and confirmed negative for PEDV, porcine deltacoronavirus (PDCoV), transmissible gastroenteritis virus (TGEV), and porcine rotaviruses (groups A, B, & C) by virus-specific PCRs on rectal swabs and negative for PEDV antibody by a virus-specific indirect fluorescent antibody (IFA) assay on serum samples. Upon arrival at the Iowa State University Laboratory Animal Resources (LAR) facilities, all pigs were administered an intramuscular injection of Excede® (Zoetis, Florham Park, New Jersey, USA) per label instructions. Pigs were randomized by weight into 7 groups of 4 pigs each, one room per group. Within each group, pigs were housed in tubs with solid dividers completely separating each of the 4 pigs from one another. Each divided portion of the tub had dedicated water sources (Fig 1). Pigs were fed a mixture of Esbilac liquid milk replacer and yogurt and had free access to water.

After 1 day acclimation (piglets were 6 days old), pigs in groups 1–7 were orogastrically inoculated with 10^−3^, 10^−4^, 10^−5^, 10^−6^, 10^−7^, and 10^−8^ dilutions of the PEDV stock and the virus-negative cell culture media, respectively, using a size 8 French feeding tube and 60ml syringe (10ml/pig) (Table 2). Piglets were evaluated daily for clinical signs of vomiting, diarrhea, lethargy, and body condition. Diarrhea severity was assessed with the following criteria by both visual observation and rectal swabbing: ‘normal’ = no diarrhea, ‘mild diarrhea’ = soft (cowpie), ‘moderate diarrhea’ = liquid with some solid content, ‘watery diarrhea’ = watery with no solid content. Rectal swabs were collected daily from each pig from 0 day post inoculation (DPI) until necropsy and were submerged into 1ml phosphate buffered saline (PBS, 1× pH 7.4) immediately after collection. Serum was collected before inoculation and at necropsy. Duration of the study was designed to be 0–7 DPI. However, per IACUC protocol, pigs with severe clinical signs were euthanized and necropsied at 4 DPI and the remaining pigs were kept through 7 DPI for necropsy (Table 3). At necropsy, intestinal tissues and contents were grossly evaluated. Fresh proximal jejunum, middle jejunum, distal jejunum, ileum, cecum and colon were collected, along with cecum content. Additionally, a portion of the three sections of jejunum, and ileum were fixed in 10% neutral buffered formalin. Rectal swabs and cecum contents were tested by aforementioned quantitative PEDV N gene-based rRT-PCR. Formalin-fixed jejunum and ileum were submitted to the ISU VDL for histopathology and immunohistochemistry examinations.

**Table 2:** Experimental Design of the neonatal and weaned pig studies.

Virus Dilution for Inoculation	Inoculum Volume	Neonatal pig study		Weaned pig study			
		Group	Pigs	Group	Pigs	Necropsy 7 DPI	Necropsy 28 DPI
10^−3^	10 ml	1	N = 4	1	N = 6	N = 3	N = 3
10^−4^	10 ml	2	N = 4	2	N = 6	N = 3	N = 3
10^−5^	10 ml	3	N = 4	3	N = 6	N = 3	N = 3
10^−6^	10 ml	4	N = 4	4	N = 6	N = 3	N = 3
10^−7^	10 ml	5	N = 4	5	N = 6	N = 3	N = 3
10^−8^	10 ml	6	N = 4	6	N = 6	N = 3	N = 3
Neg Ctl	10 ml	7	N = 4	7	N = 6	N = 3	N = 3

**Table 3:** PEDV shedding in rectal swabs of the neonatal piglets.*

Group	Inocula (10ml/pig)			Pig ID	PEDV rRT-PCR Ct values							
	Dilution	TCID_50_/ml	Ct		0 DPI	1 DPI	2 DPI	3 DPI	4 DPI	5 DPI	6 DPI	7 DPI
1	10^−3^	562	24.22	10	>45	>45	28.63	19.3	29.6	X	X	X
				32	>45	>45	17.15	15.61	21.33	X	X	X
				15	>45	22.98	17.83	19.29	17.06	X	X	X
				6	>45	>45	17.03	15.62	14.65	X	X	X
2	10^−4^	56.2	28.22	27	>45	>45	15.44	9.96	13.06	X	X	X
				7	>45	>45	27.75	25.58	19.41	X	X	X
				29	>45	39.97	22.24	24.6	17.43	X	X	X
				28	>45	>45	20.64	21.05	30.57	X	X	X
3	10^−5^	5.62	31.37	16	>45	>45	22.15	19.58	15.87	X	X	X
				3	>45	>45	21.71	21.76	18.08	X	X	X
				5	>45	>45	20.05	19.31	16.46	X	X	X
				4	>45	33.33	19.54	22.67	17.22	X	X	X
4	10^−6^	0.562	35.28	20	>45	>45	20.19	24.36	15.03	X	X	X
				18	>45	>45	12.28	14.38	15.24	X	X	X
				26	>45	>45	21.86	21.56	17.74	X	X	X
				17	>45	>45	20.12	17.27	14.81	X	X	X
5	10^−7^	0.0562	37.62	12	>45	>45	21.55	21.24	24.69	X	X	X
				25	>45	>45	18.95	24.61	22.31	X	X	X
				14	>45	>45	21.12	20.07	23.61	X	X	X
				2	>45	>45	20.15	16.87	13.73	X	X	X
6	10^−8^	0.00562	>45	9	>45	>45	>45	14.2	19.66	X	X	X
				23	>45	>45	>45	>45	>45	>45	>45	>45
				13	>45	>45	>45	>45	>45	>45	>45	>45
				8	>45	>45	>45	>45	>45	>45	>45	>45
7	Neg Ctl	0	>45	21	>45	>45	>45	>45	>45	>45	>45	>45
				24	>45	>45	>45	>45	>45	>45	>45	>45
				1	>45	>45	>45	>45	>45	>45	>45	>45
				31	>45	>45	>45	>45	>45	>45	>45	>45

*All pigs PCR positive on rectal swabs were necropsied on day 4 due to the severity of clinical signs

### Weaned pig study design

Forty-two 3-week-old pigs purchased from a conventional breeding farm were tested at the ISU VDL and confirmed negative for PEDV, PDCoV, TGEV, and porcine rotaviruses (groups A, B, & C) by virus-specific PCRs on rectal swabs and negative for PEDV by a virus-specific indirect fluorescent antibody assay. All pigs were administered an intramuscular injection of Excede® (Zoetis) per label instructions. Pigs were randomized by weight into 7 groups of 6 pigs each, one group per room, with the 6 pigs within each group housed together in one room on a solid floor.

After 3 days acclimation (pigs were 24 days old), pigs in groups 1–7 were orogastrically inoculated with 10^−3^, 10^−4^, 10^−5^, 10^−6^, 10^−7^, and 10^−8^ dilutions of the PEDV stock and the virus-negative cell culture media, respectively, using a size 10 French feeding tube and 60ml syringe (10ml/pig) (Table 2). Pigs were evaluated for clinical signs of vomiting, diarrhea, lethargy, and body condition daily for the first week, every other day for the second week and then once weekly until 28 DPI. Rectal swabs were collected at 0–7, 10, 14, 21, and 28 DPI. Serum was collected and body weight was recorded on 0, 7, 14, 21, and 28 DPI. Three of the six pigs in each group were randomly selected for necropsy at 7 DPI with the remaining 3 pigs being necropsied at 28 DPI. Sample collections at necropsy and post-mortem diagnostics were identical to those in the neonatal pigs study. In addition, serum samples were tested for PEDV antibody using virus-specific IFA assay, virus neutralization (VN) test, and a whole virus-based enzyme linked immunosorbent assay (ELISA).

### Histopathology and immunohistochemistry

All sections of formalin-fixed jejunum and ileum were microscopically evaluated by a veterinary pathologist blinded to individual animal identifications and treatment groups. Three representative villi and crypts with integrated longitudinal sections were randomly selected from ileum of each pig for measurement of villus heights and crypt depths using a computerized image system following previously described procedures [24]. Villus-height-to-crypt-depth ratio of each tissue was calculated as the quotient of the average villus length divided by the average crypt depth.

Serial sections of ileums were evaluated for PEDV antigen by immunohistochemistry (IHC) using a PEDV-specific monoclonal antibody (BioNote, Hwaseong-si, Gyeonggi-do, Korea) as previously described [24]. The IHC antigen detection was semi-quantitatively scored as previously described [26] with the following criteria: 0 = no staining; 1 = approximately 1–10% enterocytes with positive staining; 2 = approximately 10%-25% enterocytes with positive staining; 3 = approximately 25%-50% enterocytes with positive staining; 4 = approximately 50%-100% enterocytes with positive staining.

### Indirect fluorescent antibody (IFA) assay

Serum samples (N = 147) collected at 0, 7, 14, 21, and 28 DPI from the weaned pig study were tested for antibodies by the PEDV IFA assay following the previously described procedures [24] with modifications. For IFA plate preparation, confluent Vero cells grown in 96-well plates were inoculated with 100 μl/well of the U.S. PEDV prototype strain USA/IN19338/2013 at 1000 TCID_50_/ml and incubated at 37°C, with 5% CO_2_. Twenty four hours later, the inocula were removed and cells were fixed with cold 80% acetone for 10 min. Plates were air dried, sealed and stored at -80°C until use. Serum samples were serially two-fold diluted from 1:40 to 1:5120 in PBS, and 100μl of each diluted serum was added to each well of the IFA plates. After one hour incubation at 37°C, the sera were removed and plates rinsed twice with PBS. Then 60μl/well of 1:50 diluted goat anti-swine IgG (γ) antibody conjugated with FITC (Kirkegaard & Perry Laboratories, Inc., Gaithersburg, Maryland) was added to each well and incubated at 37°C for 1 h. Plates were then rinsed twice with distilled water and cell staining was examined under a fluorescent microscope. A positive signal at a serum dilution of 1:40 or higher was considered to be IFA antibody positive.

### Virus neutralization (VN) test

Serum samples (N = 147) from the weaned pig study were tested for PEDV neutralizing antibodies. Serum samples were first inactivated at 56°C for 30 min, then 2-fold serially diluted from 1:4 to 1:512 dilutions in 96-well plates with a volume of 75μl per well after dilution. Subsequently 75μl of 4 × 10^3^ TCID_50_/ml of PEDV strain USA/IN19338/2013 was mixed with the equal volume of diluted sera and incubated for 1 h at 37°C with 5% CO_2_. Then 100μl of the serum-virus mixture (containing 200 TCID_50_ of virus) was transferred to 96-well plates with a Vero cell monolayer, and the plates were incubated for 1 h at 37°C. The cells were washed twice with post-inoculation media and incubated with 100μl/well of such media for 48 h at 37°C with 5% CO_2_. Then cells were fixed with cold 80% acetone and stained with PEDV N protein-specific monoclonal antibody SD6-29 conjugated to FITC (Medgene, Brookings, South Dakota) at a 1:100 dilution for 40 min. The staining was examined under a fluorescent microscope. The reciprocal of the highest serum dilution resulting in >90% reduction of staining as compared to the negative serum control was defined as the VN titer of the serum sample. A VN titer of ≥8 was considered positive.

### Whole virus-based ELISA for PEDV antibody detection

A U.S. PEDV prototype strain whole virus-based ELISA was developed and validated at the ISU VDL for detection of PEDV-specific IgG antibody. Each batch of one liter of PEDV propagated in Vero cells (infectious titers ranging from 10^5^–10^6^ TCID50/ml) were subjected to one freeze-thaw and then centrifuged at 4,000 × *g* for 15 min to remove cell debris. The virus was then pelleted by ultracentrifugation at 140,992 × *g* for 3 h. The virus pellet was resuspended in sterile PBS (1× pH 7.4) at a ratio of 1:100 of the original volume and stored at -80°C until use. Polystyrene 96-well microtitration plates (Thermo Fisher Scientific) were coated with the viral antigen solution (100μl/well) and incubated at 4°C overnight. Plates were washed 5 times, blocked (300μl/well) with PBS containing 1% bovine serum albumin (Jackson ImmunoResearch Inc., West Grove, Pennsylvania), and incubated at 25°C for 2 h. Plates were then dried at 37°C for 4 h and stored at 4°C in a sealed bag with desiccant packs until use. Serum samples were 1:50 diluted and added to the coated plates (100μl/well). Plates were incubated at 25°C for 1 h and then washed 5 times with PBS (1× pH 7.4). Subsequently 100μl of peroxidase-conjugated goat anti-pig IgG (Fc) antibody (Bethyl Laboratories Inc., Montgomery, Texas, USA) at 1:25,000 dilution was added to each well and the plates were incubated at 25°C for 1 h. After a washing step, 100μl of tetramethylbenzidine-hydrogen peroxide substrate solution (TMB, Dako North America Inc., Carpinteria, California, USA) was added to each well. The plates were incubated for 5 min at room temperature and the reaction was stopped by adding 50μl of stop solution (1 M sulfuric acid) to each well. Reactions were measured as optical density (OD) at 450 nm using an ELISA plate reader operated with commercial software (Biotek® Instruments Inc., Winooski, Vermont, USA). The antibody response in serum samples was represented as sample-to-positive (S/P) ratios calculated as: S/P ratio = (sample OD–blank well control mean OD) / (positive control mean OD–blank well control mean OD). After multiple optimizations, the S/P ratios of >0.8 were considered antibody positive, 0.6–0.8 as suspect, and <0.6 as negative [27, 28].

### Statistics

The villus height, crypt depth, and villus/crypt ratio were compared with a generalized linear mixed (GLIMMIX) model. IHC scores were compared using a Kruskal-Wallis Test. All tests were performed using JMP software (SAS Institute, Cary, North Carolina, USA), with P<0.05 considered significant.

## Results

### Infectious titers and PCR results of the 10-fold serially diluted PEDV stock

A PEDV virus stock (USA/IN19338/2013 passage 7) with a known infectious titer of 5.62×10^5^ TCID50/ml was 10-fold serially diluted into virus-negative cell culture medium, giving rise to theoretical infectious titers of 5.62×10^5^ to 5.62×10^−5^ TCID_50_/ml for the 10^0^ to 10^−10^ dilutions, respectively (Table 1). Infectious titers of each virus dilution were back-titrated in Vero cells by end-point titration assay (TCID_50_/ml) and plaque assay (PFU/ml), with results summarized in Table 1. The stock virus and 10^−1^ to 10^−4^ dilutions all had the same logarithms of titers as the theoretical titers based on the dilution factor, regardless of titration assays in the units of TCID_50_/ml or PFU/ml. The 10^−5^ virus dilution had an infectious titer of 5 PFU/ml but remained undetected by the end-point titration assay in the unit of TCID_50_/ml. The 10^−6^ to 10^−10^ virus dilutions were undetected by either the end-point titration assay or the plaque assay during back titration.

Each virus dilution was also tested in triplicate by a quantitative PEDV N gene-based rRT-PCR. The virus stock had an average Ct of 12.2 and the Ct values increased by roughly 3–4 for every 10-fold dilution until the 10^−7^ dilution that had a Ct value of 37.62, beyond which all dilutions had Ct>45 (Table 1). This range of Ct values correlated to 2.1×10^10^ genomic copies/ml in the virus stock through 689 genomic copies/ml in the 10^−7^ dilution, and an undetected number of genomic copies/ml in all dilutions beyond that (Table 1).

The PEDV dilutions 10^−3^ to 10^−8^, having theoretical infectious titers of 5.62×10^2^ to 5.62×10^−3^ TCID_50_/ml and PCR Ct values of 24.22 to >45, together with virus-negative culture medium control, were selected to determine the infection outcomes in 5-day-old piglets and 3-week-old pigs (Table 2).

### Infection outcomes in 5-day-old piglets

All four 5-day-old piglets in the groups 1 to 5 (inoculated with virus dilutions 10^−3^ to 10^−7^) and one pig (pig ID 9) in group 6 (inoculated with virus dilution 10^−8^) developed mild to watery diarrhea at 1 DPI and diarrhea lasted through the study period. Dehydration and lethargy were also observed in these piglets at 2 DPI through the end of the study period. Vomiting was only observed in piglets inoculated with the 10^−3^ dilution (5.62×10^2^ TCID_50_/ml, 10ml/pig) at 1 DPI. Due to severity of clinical signs, these piglets were euthanized and necropsied at 4 DPI. In contrast, 3 pigs (pig IDs 23, 13 and 8) in the group 6 inoculated with virus dilution 10^−8^ and all 4 pigs in the negative control group remained active and clinically unaffected throughout the 7-day study period.

**Fig 2:**
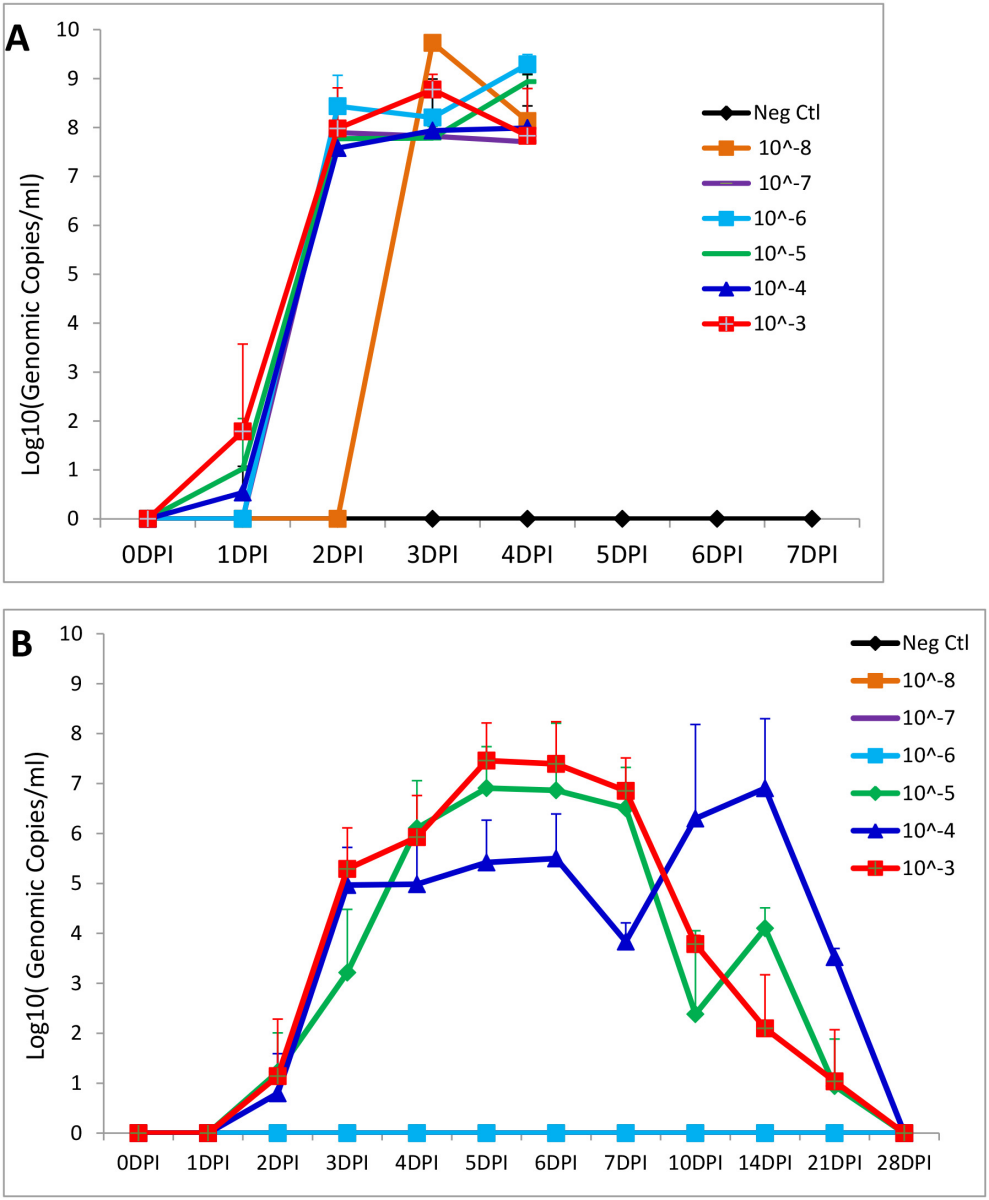
Virus shedding in rectal swabs of the 5-day-old pig study (A) and of the 3-week-old pig study (B) as determined by a quantitative PEDV N gene-based rRT-PCR. The virus titers (Log_10_Genomic copies/ml) at each time points were mean virus titers of all available pigs (both PCR positive and negative pigs) except the 10^−8^ group in the 5-day-old pig study where only one out of 4 pigs was positive in the study period and included in figure (A).

Fecal virus shedding of the inoculated 5-day-old piglets is summarized in Table 3 and Fig 2A. One pig each in groups 1, 2, and 3 (10^−3^, 10^−4^ and 10^−5^ dilutions) were PCR positive on rectal swabs at 1 DPI with Ct values 22.98–39.97. All 4 pigs in each of groups 1–5 (10^−3^–10^−7^ dilutions) were PCR positive on rectal swabs by 2 DPI and remained positive through the necropsy day 4 DPI. One pig (pig ID 9) in group 6 (10^−8^ dilution) shed virus in rectal swabs starting from 3 DPI with a Ct value of 14.2 (Table 3). The quantitative genomic copies/ml of PEDV RNA in rectal swabs is shown in Fig 2A. The average virus shedding in groups 1–5 (10^−3^ to 10^−7^ dilutions) at 2–4 DPI and virus shedding in pig ID 9 from group 6 (10^−8^ dilution) at 3–4 DPI were similar, having 10^7.6^ to 10^9.2^ genomic copies/ml. All rectal swabs from the 3 unaffected pigs (IDs 23, 13 and 8) in group 6 (10^−8^ dilution) and all 4 pigs in the negative control group were negative by PEDV PCR. Cecum contents obtained at necropsy were also tested by PEDV PCR and the results were consistent with those of the rectal swabs collected on the corresponding necropsy days (data not shown).

Thin-walled small intestine and soft to watery contents in small intestines, ceca and colons were observed at necropsy in pigs in groups 1–5 (10^−3^–10^−7^ dilutions) and one pig (ID 9) of group 6; similar villous atrophy consistent with viral infection was observed in these pigs. Villus height and crypt depth were measured, villus-height-to-crypt-depth ratios were calculated, and the magnitude of IHC staining was scored and compared on ileums of different groups (Table 4). Pigs in groups 1–5 (10^−3^–10^−7^ dilutions) were all necropsied at 4 DPI and there were no significant differences between groups in villus height, crypt depth, villus/crypt ratio, or IHC scores. Only one pig (ID 9) from group 6 was necropsied at 4 DPI and was thus not included in the statistical analysis. Microscopic intestinal lesions consistent with viral enteritis were not observed in the 3 remaining pigs in group 6 and the negative control pigs (group 7); villus height, crypt depth, and villus/crypt ratio of ileums of these pigs were not significantly different from each other; IHC staining was negative on these pigs. Compared to the pigs in group 6 (IDs 23, 13 and 8) and group 7 (negative control), the pigs in groups 1–5 had decreased villus heights, increased crypt depth, and lower villus/crypt ratios; however, statistical differences were not analyzed because they were necropsied at different DPI.

**Table 4:** Mean villus height (μm), crypt depth (μm), villus/crypt ratio, and IHC scores in sections of ileum from neonatal piglets.

	Group	Dilution	Pigs	Villous Height	Crypt Depth	Villus/Crypt ratio	IHC Score
				Mean ± SEM	Mean ± SEM	Mean ± SEM	Mean ± SEM
Necropsy at 4 DPI	1	10^−3^	N = 4	218.13 ± 16.33	161.64 ± 6.81	1.35 ± 0.10	2.94 ± 0.68
	2	10^−4^	N = 4	208.56 ± 39.94	171.22 ± 6.59	1.21 ± 0.21	3.81 ± 0.19
	3	10^−5^	N = 4	272.19 ± 22.03	162.21 ± 9.71	1.67 ± 0.04	3.88 ± 0.13
	4	10^−6^	N = 4	230.85 ± 19.33	152.46 ± 4.52	1.51 ± 0.12	3.63 ± 0.24
	5	10^−7^	N = 4	228.18 ± 33.57	158.24 ± 10.89	1.45 ± 0.21	3.94 ± 0.06
	6*	10^−8^	N = 1	222.49	143.12	1.55	4.00
Necropsy at 7 DPI	6	10^−8^	N = 3	423.17 ± 51.35	118.60 ± 16.81	3.81 ± 0.87	0.00 ± .000
	7	Neg Ctl	N = 4	450.76 ± 20.05	108.40 ± 12.61	4.31 ± 0.46	0.00 ± 0.00

* Mean and standard error of the mean (SEM) could not be calculated nor could statistical comparison be performed on the one pig from Group 6 necropsied at 4 DPI.

### Infection outcomes in 3-week-old pigs

In groups 1–3 (10^−3^ to 10^−5^ dilutions), 1/6, 1/6, and 6/6 pigs in each group developed mild diarrhea at 2, 3, and 4–7 DPI; mild dehydration or lethargy was observed in a few pigs in these groups during 3–7 DPI. No diarrhea, dehydration, or lethargy was observed in pigs in groups 4–7 (10^−6^ to 10^−8^ dilutions and negative control) throughout the duration of the study.

Fecal virus shedding from the inoculated 3-week-old piglets is summarized in Table 5 and Fig 2B. Pigs in all groups were PCR negative on rectal swabs collected before inoculation and at 1 DPI. One pig each in groups 1 and 2 along with two pigs in group 3 were PCR positive on rectal swabs by 2 DPI. All pigs in groups 1–3 were PEDV PCR positive by 4 DPI and remained positive through at least 7 DPI although there were variations on PCR Ct values between individual pigs. Most of the remaining pigs in groups 1–3, after necropsy at 7 DPI, continued to shed virus in rectal swabs during 10–21 DPI but all pigs in these groups ceased shedding virus at 28 DPI (Table 5). Based on the quantitative genomic copies/ml of PEDV RNA in rectal swabs, the average virus shedding in groups 1–3 (10^−3^ to 10^−5^ dilutions) had similar patterns: increasing virus shedding during 2–6 DPI and decreasing virus shedding after 7 DPI (Fig 2B). However, pigs in group 2 (10^−4^ dilution) had a peak virus shedding during 10–14 DPI (Fig 2B). All rectal swabs from pigs in groups 4–7 (10^−6^ to 10^−8^ dilutions and negative control) were negative by PEDV PCR through the study period. Cecum contents obtained at necropsy were also tested by PEDV PCR in all pigs and the results were consistent with those of the rectal swabs collected on the corresponding necropsy days (data not shown).

**Table 5:** PEDV shedding in rectal swabs of the weaned pigs.*

Group	Inocula (10ml/pig)			Pig ID	PEDV rRT-PCR Ct values											
	Dilution	TCID_50_/ml	Ct		0 DPI	1 DPI	2 DPI	3 DPI	4 DPI	5 DPI	6 DPI	7 DPI	10 DPI	14 DPI	21 DPI	28 DPI
1	10^−3^	562	24.22	7	>45	>45	>45	34.88	34.87	29.42	31.10	32.94	33.30	>45	>45	>45
				66	>45	>45	>45	32.09	19.24	19.72	15.62	19.17	34.60	35.21	36.71	>45
				89	>45	>45	24.00	16.66	20.13	15.04	20.08	22.26	35.33	37.94	>45	>45
				3	>45	>45	>45	35.01	34.60	29.67	30.79	24.69	X	X	X	X
				10	>45	>45	>45	27.50	24.34	20.99	19.13	26.88	X	X	X	X
				86	>45	>45	>45	29.64	29.63	16.71	16.14	17.97	X	X	X	X
2	10^−4^	56.2	28.22	18	>45	>45	>45	32.68	34.18	30.13	32.26	35.07	24.21	14.46	34.12	>45
				64	>45	>45	>45	33.44	33.92	31.83	32.25	30.72	15.70	29.83	36.08	>45
				96	>45	>45	31.05	17.61	16.78	14.52	13.78	30.43	37.68	27.13	35.69	>45
				77	>45	>45	>45	33.35	31.69	32.18	31.73	38.46	X	X	X	X
				83	>45	>45	>45	32.14	33.61	32.19	28.42	34.73	X	X	X	X
				93	>45	>45	>45	33.13	31.81	32.29	33.07	36.02	X	X	X	X
3	10^−5^	5.62	31.37	12	>45	>45	>45	>45	34.16	28.73	30.51	21.96	28.21	30.87	37.67	>45
				74	>45	>45	33.07	19.19	19.22	17.96	17.29	32.14	>45	35.71	>45	>45
				90	>45	>45	36.53	32.05	19.58	15.30	16.47	17.87	34.13	33.42	>45	>45
				2	>45	>45	>45	37.58	33.87	31.46	42.98	35.07	X	X	X	X
				78	>45	>45	>45	34.72	19.07	19.75	13.03	21.25	X	X	X	X
				99	>45	>45	>45	>45	33.23	29.56	23.39	22.69	X	X	X	X
4^‡^	10^−6^	0.562	35.28	6; 68; 81	>45	>45	>45	>45	>45	>45	>45	>45	>45	>45	>45	>45
				9; 14; 91	>45	>45	>45	>45	>45	>45	>45	>45	X	X	X	X
5^‡^	10^−7^	0.0562	37.62	15; 20; 67	>45	>45	>45	>45	>45	>45	>45	>45	>45	>45	>45	>45
				11; 16; 98	>45	>45	>45	>45	>45	>45	>45	>45	X	X	X	X
6^‡^	10^−8^	0.00562	>45	4; 21; 87	>45	>45	>45	>45	>45	>45	>45	>45	>45	>45	>45	>45
				8; 73; 79	>45	>45	>45	>45	>45	>45	>45	>45	X	X	X	X
7^‡^	Neg Ctl	0	>45	70; 76; 92	>45	>45	>45	>45	>45	>45	>45	>45	>45	>45	>45	>45
				17; 72; 88	>45	>45	>45	>45	>45	>45	>45	>45	X	X	X	X

* 3 pigs were necropsied at 7 DPI with the remaining 3 being necropsied at 28 DPI.

^‡^ For groups 4, 5, 6, and 7, rectal swabs from all pigs were negative by PEDV rRT-PCR. To save space, 3 pigs are shown in one row.

For pigs necropsied at 7 DPI, microscopic lesions consistent with viral infection were observed in 2/3 pigs of group 1 (10^−3^ dilution), 1/3 pigs of group 2 (10^−4^ dilution), 1/3 pigs of group 3 (10^−5^ dilution), and 0/3 pigs of groups 4–7 (10^−6^ to 10^−8^ dilutions and negative control); IHC staining results were consistent with the presence or absence of microscopic lesions. However, villus heights, crypt depths, villus/crypt ratios of pigs in all groups 1–7 were not significantly different from each other at 7 DPI (data not shown). For pigs necropsied at 28 DPI, no microscopic lesions or IHC staining were observed in any pigs of groups 1–7.

**Fig 3:**
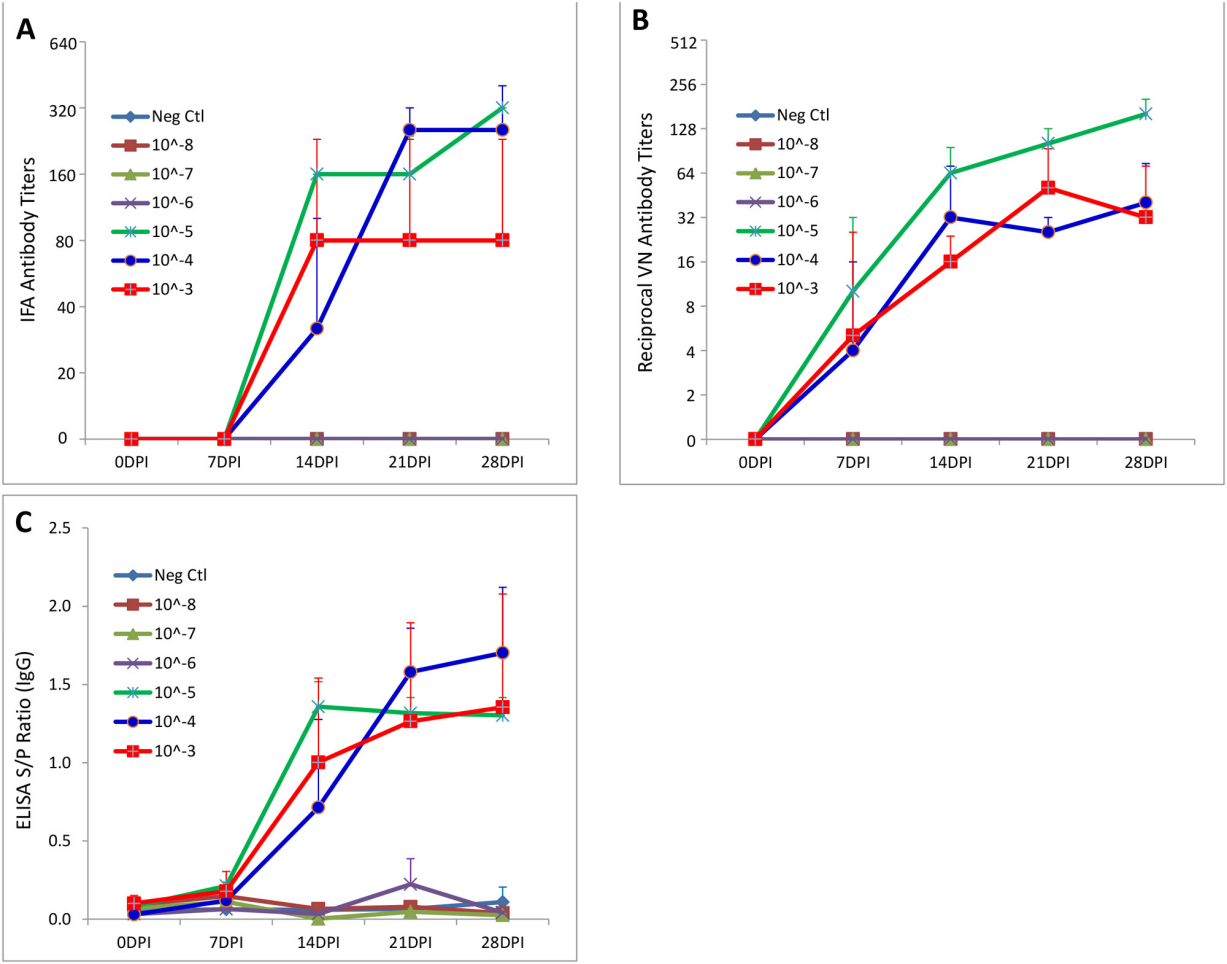
Serum antibody responses in 3-week-old pigs inoculated with serial dilutions of PEDV. IFA antibody titers (A), serum virus neutralizing antibody titers (B), and PEDV whole virus-based ELISA antibody titers (C) were shown for 3 pigs in each group that went through 28 days post inoculation.

Seroconversion was first observed at 14 DPI for pigs in groups 1–3 (10^−3^ to 10^−5^ dilutions) by both IFA (Fig 3A) and whole virus-based ELISA (Fig 3C) assays and then remained through 28 DPI. PEDV neutralizing antibodies was first detected by VN test at 7 DPI in 1/3 pigs of groups 1 and 2, and in 2/3 pigs of group 3 (Fig 3B). Serum anti-PEDV antibody was not detected in pigs from groups 4–7 (10^−6^ to 10^−8^ dilutions and negative control) throughout the study period by any of the 3 antibody assays.

## Discussion

One objective of this study was to determine the correlation of PEDV PCR Ct values to the infectious titers. A U.S. PEDV prototype strain cell culture isolate USA/IN19338/2013 was 10-fold serially diluted and tested in this study. Since there are variations among laboratories in reporting virus titer units, we determined the infectious titers of the PEDV dilutions using both end-point titration assay and plaque assay. It was found that one TCID_50_/ml is almost equivalent to one PFU/ml for PEDV. Based on a quantitative PEDV N gene-based rRT-PCR we developed, PEDV with an infectious titer of 5 TCID_50_/ml or 5 PFU/ml had a Ct value of approximate 31 corresponding to about 4.8×10^4^ genomic copies/ml. An equation of X = 10^(Ct-47.262)/-3.3969^, where X = genomic copies/ml, was used to transform the Ct values into estimated genomic copies of PEDV RNA per ml in samples. However, these results need to be interpreted with caution. First, a PEDV N gene-based primers and probe and reagents from the Path-ID™ Multiplex One-Step RT-PCR Kit (Thermo Fisher Scientific) were used in this study. PEDV rRT-PCRs using other primers, probe, and master mix buffer reagents may generate results somewhat different from what we obtained in this study. Second, correlations of the infectious titers, Ct values and genomic copies/ml in this study were obtained using a PEDV cell culture isolate diluted in cell culture medium. For PEDV present in other sample matrices, this correlation could have some variations.

Another objective of this study was to determine the minimum infectious dose of PEDV in 5-day-old and 3-week-old naïve conventional pigs. In 5-day-old pigs, 100% of pigs (4/4 pigs) became infected with 10ml of inoculum having titers 560–0.056 TCID_50_/ml (Ct 24.2–37.6); 25% of pigs (1/4 pigs) became infected with 10ml of inoculum having titer 0.0056 TCID_50_/ml (Ct>45) (Table 6). In 3-week-old pigs, 10ml of inoculum with titers 560–5.6 TCID_50_/ml (Ct 24.2–31.4) resulted in 100% (6/6 pigs) infection while 10ml of inoculum with titers 0.56–0.0056 TCID_50_/ml (Ct values 35.3 –>45) could not establish infection in any pigs under these study conditions (Table 6). The smallest virus magnitude that can cause infection in at least one pig in a group is considered the minimum infectious dose. In the 5-day-old pig study, 4 pigs in each group were housed separately without direct contact between pigs. The minimum infectious dose of PEDV was 0.056 TCID_50_ or lower in 5-day-old pigs. In the 3-week-old pig study, due to space limitation, it was not possible to house all pigs individually; however, this did not preclude determination of the minimum infectious dose in the current study because the weaned pig groups 4–6 (dilutions 10^−6^ to 10^−8^) had no single pig positive during 28 days study periods. However, if conditions allowed, for minimum infectious dose studies, pigs should ideally be housed separately during long-term study periods to avoid possible pig-to-pig transmission. The MID of PEDV was 56 TCID_50_ in 3-week-old pigs under our study conditions. This suggests that the MID for neonatal pigs is at least 1000-fold lower than it is in weaned pigs.

**Table 6:** Summary of PEDV infection outcomes in neonatal and weaned pigs.

Group	Inocula (10ml/pig)			Infection Outcomes	
	Dilution	TCID_50_/ml	Ct	Neonatal Piglets (5-day-old)	Weaned Pigs (21-day-old)
1	10^−3^	562	24.22	100% (4/4)	100% (6/6)
2	10^−4^	56.2	28.22	100% (4/4)	100% (6/6)
3	10^−5^	5.62	31.37	100% (4/4)	100% (6/6)
4	10^−6^	0.562	35.28	100% (4/4)	0 (0/6)
5	10^−7^	0.0562	37.62	100% (4/4)	0 (0/6)
6	10^−8^	0.00562	>45	25% (1/4)	0 (0/6)
7	Neg Ctl	0	>45	0 (0/4)	0 (0/6)

Our study confirms that 5-day-old pigs are more susceptible than 3-week-old pigs to PEDV infection. PEDV progresses faster, the fecal virus shedding level is higher, and disease is more severe, in 5-day-old pigs than in 3-week-old pigs (Fig 2A and 2B). We also observed that the response among four 5-day-old pigs in each group was comparable as reflected by similar virus shedding levels (Table 3) and similar microscopic lesions. In contrast, there was more variation in virus shedding among six 3-week-old pigs in each group (Table 3). These observations were consistent with previous reports on age-dependent resistance of pigs to TGEV infection [29]. However, in either 5-day-old or 3-week-old pig models, once pigs were infected and virus replication began, the initial dose of virus appears to have little impact at the group level on the average amount of fecal viral shedding, average severity of microscopic lesions/IHC staining, or the average magnitude of antibody titer that is subsequently developed. It should be noted that the 5-day-old piglets in this study were fed a mixture of liquid milk replacer and yogurt, which were possibly devoid of IgG, IgA and other immunologic ingredients found in sow milk. Thus, the piglets raised under the current study conditions could be more susceptible to PEDV infection than piglets at the corresponding ages that were naturally nursed by sows under field conditions.

Previous studies also demonstrated that PEDV infection induces greater disease severity in neonatal piglets than in weaned pigs although the minimum infectious dose of PEDV was not determined in those studies [24, 30–32]. The exact reasons for the greater severity of PED in neonatal piglets are unknown but there may be several contributing factors including: 1) an immature immune system in neonatal piglets [33, 34]; for example, the level of natural IFN-α production by porcine blood mononuclear cells is lower in neonates and the phagocytic cells present in newborn piglets generally have reduced phagocytic activity as compared with adult animals [35, 36]; 2) neonatal piglets are more vulnerable to dehydration and the electrolyte and fluid imbalance [33]; 3) the intestinal villi of neonatal piglets are longer and may have more mature permissive enterocytes than weaned pigs [37]; 4) slower replacement of villous enterocytes (7–10 days) in neonatal pigs compared to 2–4 days in weaned pigs [38]. The virus mainly infects and destroys mature enterocytes lining the villi of small intestine, resulting in shortening and blunting of villi. But the intestinal crypt epithelial cells basically remain uninfected and serve to replace the destroyed villous enterocytes. Regeneration of villous enterocytes in neonatal piglets is not as rapid as that in weaned pigs; this may explain why villous atrophy is more severe in neonatal than in weaned pigs. Recently Jung et al [31] further demonstrated that neonatal pigs have low numbers of LGR5+ (marker for crypt stem cell) cells and low levels of Ki67 staining (marker for crypt proliferation) in contrast to large numbers of LGR5+ cells in the crypts and high proliferation of intestinal crypt cells in weaned pigs, explaining the rapid turnover or recovery rate of villous enterocytes in weaned pigs.

Success rate of PEDV isolation in cell culture has been low. Currently swine bioassay has remained the most reliable means to determine if infectious PEDV is present in a sample. Data from this study suggests a neonatal pig bioassay is more sensitive than a weaned pig bioassay to assess PEDV infectivity. Regarding duration of the swine bioassay, we observed that one pig (ID 9) in group 6 of the 5-day-old pig study did not shed virus in rectal swabs until 3 DPI; thus 2-day duration of pig bioassay may not be sufficient to evaluate the infection outcome. We propose that 7-day duration would be considered appropriate for PEDV swine bioassay.

In summary, using a U.S. PEDV prototype isolate diluted in culture medium, we determined the correlations of PEDV infectious titers to PCR Ct values and genomic copies per ml. We further demonstrated that PEDV infectious dose is age-dependent with a significantly lower MID for neonatal pigs than for weaned pigs. This information should be taken into consideration when interpreting clinical relevance of PEDV PCR results and when designing a PEDV bioassay model. The observation of such a low MID in neonates also emphasizes the importance of strict biosecurity and thorough cleaning/disinfection on sow farms. In this study, the effect of PEDV infectious doses on infection outcomes was evaluated in naïve conventional neonatal and weaned pigs. It must be noted that infectious dose effects in previously exposed or vaccinated pigs may be different from naïve pigs. In addition, a U.S. PEDV prototype cell culture isolate was used in this study and it remains to be determined whether or not U.S. PEDV S-INDEL-variant isolate has similar minimum infectious dose and pathogenicity.
